# Developing the Workforce of the Digital Future: mHealth Competency and Fidelity Measurement in Community-Based Care

**DOI:** 10.1007/s41347-024-00385-y

**Published:** 2024-01-13

**Authors:** Sarah L. Kopelovich, Benjamin E. Buck, Justin Tauscher, Aaron R. Lyon, Dror Ben-Zeev

**Affiliations:** Department of Psychiatry & Behavioral Sciences, University of Washington School of Medicine, 1959 NE Pacific Street, Box 356560, Seattle, WA 98195-6560 USA

**Keywords:** Competencies, Fidelity, Clinical quality assurance, Mobile health, Digital mental health

## Abstract

Integrating mobile health (mHealth) interventions into settings that serve diverse patient populations requires that prerequisite professional competencies are delineated and that standards for clinical quality assurance can be pragmatically assessed. Heretofore, proposed mHealth competencies have been broad and have lacked a framework to support specific applications. We outline the meta-competencies identified in the literature relevant to mHealth interventions and demonstrate how these meta-competencies can be integrated with population- and intervention-related competencies to help guide a pragmatic approach to competency assessment. We present a use case based on *FOCUS*—an evidence-based mHealth intervention designed for individuals with serious mental illness and currently being implemented in geographically and demographically diverse community behavioral health settings. Subsequent to identifying the cross-cutting competencies relevant to the target population (outpatients experiencing psychotic symptoms), substratal intervention (Cognitive Behavioral Therapy for psychosis), and treatment modality (mHealth), we detail the development process of an mHealth fidelity monitoring system (mHealth-FMS). We adhered to a published sequential 5-step process to design a fidelity monitoring system that aligns with our integrated mHealth competency framework and that was guided by best practices prescribed by the Treatment Fidelity Workgroup of the National Institutes of Health Behavior Change Consortium. The mHealth-FMS is intended to enhance both clinical and implementation outcomes by grounding the mHealth interventionist and the system of care in which they operate in the core functions, tasks, knowledge, and competencies associated with system-integrated mHealth delivery. Future research will explore acceptability and feasibility of the mHealth-FMS.

The mounting empirical support for digital health interventions coupled with the imperative to digitize interventions during the global COVID-19 pandemic has hastened demand for digital health technologies that can be integrated in routine care settings (Ben-Zeev, 2020). When integrated into an individual’s care, mobile health (mHealth) technologies can enable in vivo rehearsal of therapeutic techniques, information exchange with a patient’s care team, and provide momentary assessment of psychological and biological metrics that can enhance insights into treatment response. Indeed, emerging data suggests that some mHealth interventions for serious mental health conditions can achieve outcomes that are at least as effective as clinic-based services (Ben-Zeev et al., 2018), while increasing client engagement (Buck et al., 2020) and reducing costs (Ben-Zeev et al., 2021).

The mHealth growth spurt has propagated both interest and investments in mHealth across healthcare systems. Consequently, scientific and policy communities are grappling with pragmatic and empirical questions related to mHealth adoption and integration strategies. Research on the efficacy, effectiveness, and implementation of clinician-supported digital applications designed for individuals with mental illness supports the use of a digital interventionist in the administration of mHealth and its integration into treatment plans and delivery (Ben-Zeev et al., 2015; Mohr et al., 2021; Schueller et al., 2016; Wisniewski & Torous, 2020; Wisniewski et al., 2020). A recent meta-systematic review, which analyzed the results of 31 meta-analyses consisting of 505 independent trials (Werntz et al., 2023), found larger effect sizes among digital mental health intervention trials that included human-supported mHealth interventions. Moreover, the meta-systematic review concluded that human-supported mHealth interventions may be more effective than unsupported interventions for individuals with greater symptom severity. As such, we assert that mHealth interventionists represent a new segment of the behavioral health workforce, particularly in settings that serve individuals with serious mental health conditions.

This paper, which is part of the Special Issue on Telebehavioral Health Education and Training and serves as a companion to the article, “Developing the workforce of the digital future: Leveraging technology to train community-based mental health specialists” (Buck et al., 2022), is intended to contribute to an evolving set of professional standards for mHealth interventionists. Building on our previous proposal for training community-based mHealth interventionists, we outline a competency framework for mHealth interventions that are facilitated by human support. Competency frameworks provide a structured approach to delineating the skills and knowledge needed for successful job performance (Baczyriska et al., 2016) and are therefore facilitative of fidelity and other quality monitoring efforts. We present an example of an integrative competency framework for an mHealth psychosocial intervention for serious mental illness and illustrate how the framework informed the stepwise development of a bespoke mHealth Fidelity Monitoring System (mHealth-FMS). To our knowledge, this mHealth-FMS represents a first-of-its-kind pragmatic approach to mHealth quality assurance.

## Meta-Competencies for mHealth Delivery

Multiple competency lists pertaining to health technologies have been proposed across physical and behavioral healthcare over the past decade. Many are derivatives of the US Department of Defense’s Mobile Health Training Program (MHTP; Armstrong et al., 2018), which identified five *meta-competencies* for mobile health practice. Meta-competencies are higher-order competencies that permeate all areas of practicing an intervention; they consist of skills and techniques that are considered critical to the fluent delivery of an intervention, including the decision-making involved in the execution of macro-competencies. The MHTP-identified meta-competencies include (1) an understanding of an mHealth app’s evidence base; (2) integration of mHealth into the clinical setting, consisting of workflow integration, introducing the app into clinical care, prescribing the app within a treatment plan, reviewing the collected data with the patient, and documenting the dose and response in the medical record; (3) awareness and action to protect security and privacy; (4) awareness and mitigation of ethical issues in mHealth, including informed consent on mHealth use, communication of electronic communication guidelines, and engaging in professional development to develop and maintain competencies related to mHealth use; and (5) enhancing awareness of cultural differences and awareness of potential biases that can significantly impact patient outcomes.

Subsequent scholarship on the competencies considered to be critical to delivering mHealth interventions have built on the MHTP competency framework (e.g., Hilty et al., 2020; Schueller et al., 2022) and have extrapolated core competency standards proposed for behavioral health professionals to 51 behavioral objectives and 149 measurable outcomes (Cavanagh et al., 2022). Attempts to standardize minimum training in digital health technologies among healthcare providers have already been observed in some US states (e.g., Commonwealth of Massachusetts, 2019; WA State SB 6061, 2020), but, to date, training standards—to the extent that they exist for this emerging treatment modality—have focused almost exclusively on broad and general mHealth competencies and fail to incorporate the underlying clinical skills that may be needed to deliver the intervention effectively.

## The Case for Routine Assessment of mHealth Treatment Fidelity

Treatment fidelity concerns the extent to which a delivered intervention includes the requisite components (adherence) and how skillfully those components are delivered (competence). The competence of an interventionist can be more challenging to reliably ascertain compared to what the practitioner is or is not doing. Moreover, an interventionist may know *what* to do but lack the skillfulness to execute the intervention effectively. There is a reciprocal benefit between fidelity assessment and competency development, as fidelity data can be fed back to the interventionist to support targeted practice change. In addition, assessment of fidelity supports high-quality treatment delivery in routine healthcare settings, as poor treatment fidelity can enervate treatment effects in both controlled and real-world settings (Gearing et al., 2011). It therefore stands to reason that establishing mHealth intervention fidelity standards and assessment methods can enhance the effectiveness of mHealth-delivered interventions in the real world. In addition, establishing fidelity standards can advance empirical investigations and treatment development efforts in three key ways. First, fidelity assessment requires that intervention components are operationalized to ascertain the degree to which an intervention is delivered as intended (Breitenstein et al., 2012). Thus, fidelity standards help interventionists, researchers, and treatment developers enumerate the goals and proposed mechanisms of action of the mHealth intervention. Second, fidelity assessment enables actionable, corrective feedback at the individual practitioner level (Bearman et al., 2017), thereby supporting practice improvement and enabling targeted supervision and/or continuing education. Third, fidelity assessment enables system-level intervention adoption, sustainment, program evaluation, and quality monitoring (McHugo et al., 2007; Schoenwald, 2011).

Accurate and reliable methods of measuring fidelity are critically important (Akiba et al., 2022; Mettert et al., 2020). Proctor and colleagues (2011) suggest that fidelity monitoring is among the most critical factors to implementation. Accordingly, members of both scientific and policy communities have strongly advocated for leveraging evidence-based frameworks and implementation strategies to support mHealth integration (Lipschitz et al., 2019; Mohr et al., 2021). Yet historically, fidelity assessments have been deployed in clinical trial research using methods that are both costly and impractical in routine care settings (Durlak & DuPre, 2008). Conceptualizations of and pragmatic approaches to mHealth fidelity monitoring remain underdeveloped and largely untested (Hermes et al., 2019).

## Methods

**Fig. 1:**
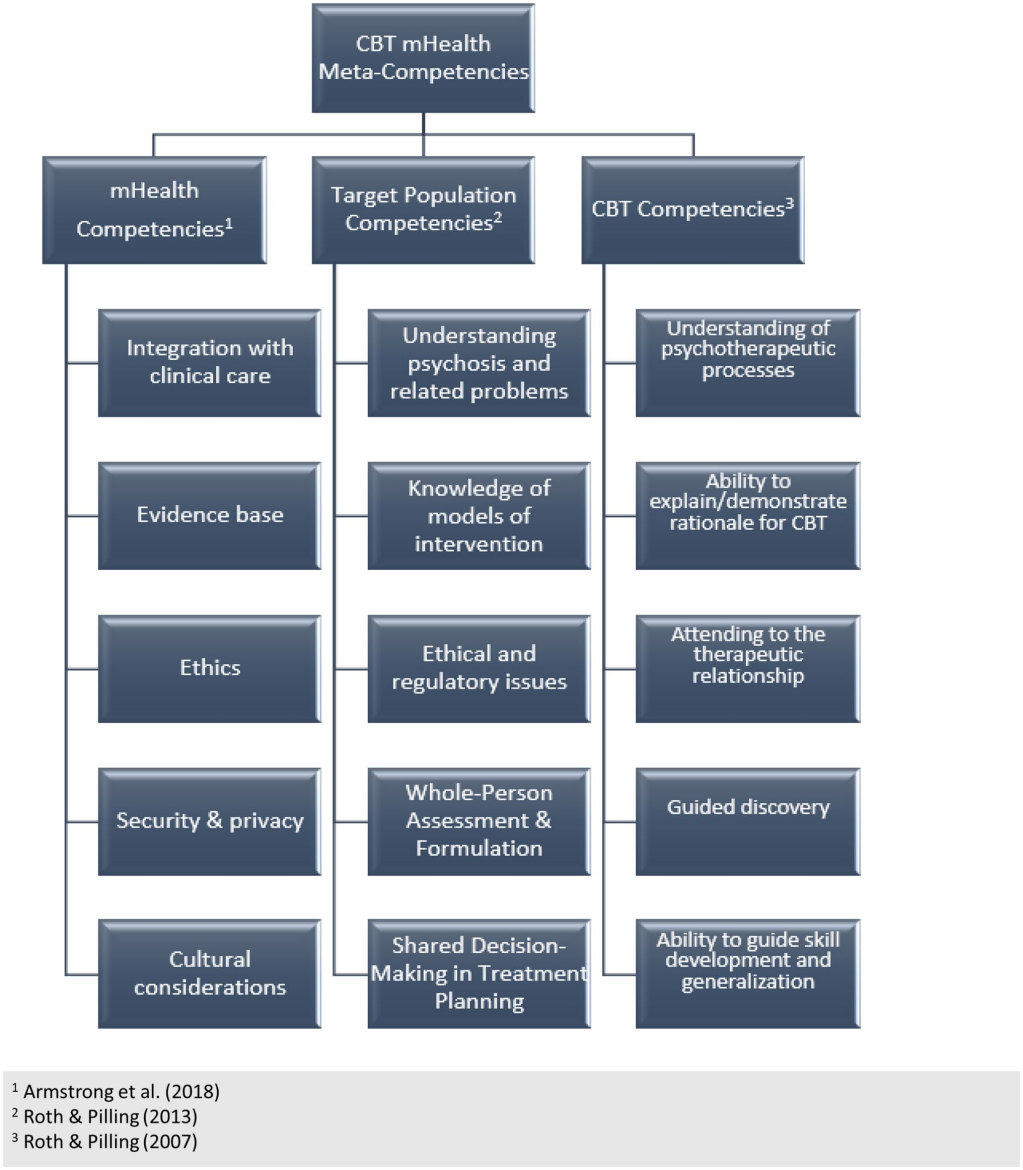
Integrative meta-competencies for the FOCUS mHealth application

We undertook this fidelity development process in the context of an NIH-funded hybrid type 3 implementation-effectiveness trial testing a digital health intervention, FOCUS, in 20 geographically and demographically diverse public behavioral health clinics (ClinicalTrials.gov NCT 04147897). Our team identified core meta-competencies associated with mHealth interventionists in the peer-reviewed and professional literature. We then engaged in a stepwise process to identify and synthesize those meta-competencies for the intervention and its target population—individuals with serious mental illness (Fig. 1; Armstrong et al., 2018; Roth & Pilling, 2007, 2013). The fidelity tool development process was guided by best practices prescribed by the Treatment Fidelity Workgroup of the National Institutes of Health Behavior Change Consortium (Bellg et al., 2004) and subsequently expounded upon by Feely and colleagues (2018). This five-step process involves (1) determining the purpose and scope of the tool, (2) identifying its essential components, (3) developing the measurement tool, (4) monitoring fidelity to the intervention, and (5) using fidelity ratings in analyses. Results explicate our application of the 5-step process to the FOCUS digital health intervention. Finally, we delineate requisite practices for the designated mHealth interventionist to ensure the competent delivery of the intervention to the service user and their primary clinician and recommend standardized practices across mHealth interventionists.

## Digital Health Intervention: FOCUS

FOCUS is an evidence-based mHealth intervention intended to complement clinic-based services for individuals with a serious mental illness. Empirical evidence supports the intervention’s acceptability and high engagement among service users (Ben-Zeev et al., 2014, 2016), its effectiveness in reducing psychiatric symptom severity and improving recovery (Ben-Zeev et al., 2018, 2019), and cost efficiency relative to clinic-based care (Ben-Zeev et al., 2021). The FOCUS intervention has three parts: (1) a client-facing smartphone application, (2) an mHealth support specialist (mHSS) who provides technical and clinical support, and (3) a practitioner dashboard that summarizes participants’ FOCUS data to enable integration with clinic-based interventions. The FOCUS application delivers content relevant to five broad treatment domains: coping with auditory hallucinations, mood difficulties (i.e., depression, anxiety), social functioning, medication use, and sleep. Participants access content via prompted notifications that ask them to check-in or on-demand. Content is informed by Cognitive Behavioral Therapy for psychosis (CBTp) interventions such as anxiety management strategies, behavioral activation, and evaluating automatic appraisals, and illness management interventions such as behavioral tailoring and sleep hygiene (AHRQ, 2017; APA, 2020). These interventions aim to support illness self-management practices and integration of cognitive-behavioral change strategies in daily life to maximize both utility and proficiency. The clinician dashboard supports continuous monitoring of self-administered interventions as well as response to treatment.

## Results

### Step 1: Determine Purpose and Scope

Although the immediate application of an mHealth fidelity monitoring system was to ensure that mHSS practices and quality assurance metrics were standardized during the course of the trial, the development team also sought to meet the broader need in the emerging landscape of digital health technology integration with traditional human-delivered care. We sought to develop a fidelity monitoring system that could be applied to the FOCUS mHealth interventionist role, was feasible and pragmatic in routine care settings such as community behavioral health clinics and was general enough to support quality assurance or research of other clinician-facilitated mHealth interventions. Specifically, we posited that ongoing use of the FMS could help operationalize quality assurance as well as empirical processes and outcomes by highlighting areas where remediation was necessary in the areas of direct service delivery or clinic-level management tasks.

Unlike traditional clinical interventionists, mHSS are more likely to need to interface with multiple stakeholders to effectively integrate mHealth into the patient’s broader treatment and ensure that security and privacy concerns have been considered by the healthcare organization. Fidelity assessment should therefore capture both intervention and implementation-related activities across stakeholder groups (e.g., patients, clinical care team, and/or organizational leadership). For instance, the mHealth interventionist provides support to clients who have been referred to the intervention. Client-facing activities include mHealth application and installation, technology troubleshooting, goal setting, motivational enhancement, and being able to explain, model, and personalize the skills demonstrated within the app. Second, mHSS are charged with enhancing uptake of the FOCUS intervention at the clinic setting among supervisors, peers, and allied clinicians. Such activities, which broadly fall into a practice facilitation role (Siantz et al., 2021), may include enhancing awareness of the FOCUS intervention, encouraging integration of FOCUS into routine care, making data available and digestible to the primary clinician or clinical team, and making data-informed recommendations for treatment planning. In recognition of the broader goal of practice facilitation to enhance uptake of the intervention within the healthcare system, clinician-facing activities also encompass elicitation of leadership engagement.

The FOCUS-FMS is intended to provide a reference point for assessing competencies in the delivery of a variety of support calls between clients and mHealth support specialists (e.g., technical coaching, clinical coaching, assessing practical versus psychological barriers to use). Given the limited duration of a typical FOCUS coaching encounter between a client and their assigned mHealth support specialist (approximately 5–15 min) and mode of administration (typically phone-based check-in), an essential feature of the fidelity monitoring process is that competencies can be discerned quickly across different types of encounters.

### Step 2: Identify the Essential Components

The specifications of the intervention should inform the most salient targets of fidelity assessment (Schoenwald et al., 2011). We endeavored to align fidelity items with the aforementioned meta-competencies associated with mHealth delivery (Armstrong et al., 2017), psychosocial interventions for patients experiencing psychosis (Roth & Pilling, 2013), and CBT (Roth & Pilling, 2007). These competencies served as the foundation for the components of the fidelity system and were mapped on to the tasks associated with the mHealth interventionist role; these include (1) technology-focused activities, such as introducing clients to the app, holding phone-based technology check-ins, and providing ongoing assistance and troubleshooting; (2) clinical activities, such as agenda setting, goal setting, goal tracking, coaching clients on the integration of FOCUS into daily routines, educational and motivational enhancement strategies, and developing follow-forward plans for using the tool independently; and (3) practice facilitation activities, including elicitation of buy-in from organizational leadership, informing leadership of practical impediments to deploying FOCUS, and communicating patient-entered data back to a clinical team in a timely, comprehensible, and clinically relevant way.

Once the competencies and core domains of activities were identified, we drafted fidelity items that aligned with core activities and competencies, both of which are described below. Given the varied tasks of the mHealth interventionist and the FMS development team’s investment in the practical utility of this system in routine care settings, the FOCUS-FMS is intended to incorporate components best-suited to address clinical interactions directly (e.g., direct observation or role play, e.g., Perepletchikova et al., 2007) as well as to adhere to a list of pre-determined tasks. In addition to serving the function of assessing and monitoring intervention integrity, the fidelity tool can also be used to train the mHSS in the mHealth intervention, to guide self-reflection, and to minimize intervention drift. Finally, the fidelity tool is able to clearly identify competencies that are necessary to measure as part of the digital interventionist role.

### Step 3: Develop the Fidelity Measurement Tool

Once the core fidelity components were established, the FOCUS-FMS development team categorized those items that were conducive to an adherence assessment and those that were conducive to a dimensional assessment of clinical competencies.

#### Adherence Assessment

Adherence to the mHealth intervention protocol is assessed through tracking quantifiable interventionist behaviors, such as the number of access points to client data, distribution of reports to the clinical team, and correspondence of data derived from the dashboard to treatment targets. The adherence checklist aims to ensure that the mHealth support specialist is completing specific predetermined tasks that facilitate uptake of the mHealth intervention at the clinical site and for the individual client. Accordingly, it includes tasks that involve clinic administrators, clinicians, and clients, as well as the data dashboard. Each item is discretely coded as present (1) or absent (0). In most circumstances, the form will be completed by the mHSS and may be cross-checked by an independent rater. In order to facilitate an accurate account of these activities, the mHSS documents practice facilitation as well as client-facing activities for actively enrolled FOCUS clients. Each set of items is designed to be rated regardless of the particular nature of the FOCUS encounter (e.g., technical coaching, clinical coaching, and/or practice facilitation). To determine the final rating, raters simply sum the number of items coded as present. Thus, if three of the five items in this section were coded as present, then the total adherence score would be *3*. Further details related to how the first Section of the FOCUS-FMS has been or could be utilized in practice are detailed in Step 4.

#### Competency Assessment

The Competency Rating Form is organized by each of the elements of an mHealth coaching encounter (as described in Buck et al., 2022) and is further broken down into micro-skills that enable competent execution of the session elements. Competencies were distilled to include (1) rapport-building and assessment; (2) agenda and pacing; (3) use of interpersonal, educational, motivational, and problem-solving strategies to effectively support clients; (4) developing goals and establishing goal-consistent behaviors; and (5) establishing a concrete and specific action plan for follow-up mHealth support. Each of the 10 items is rated on a 0–4 Likert scale (0 = incompetent execution or inappropriate omission; 1 = novice; 2 = competent; 3 = proficient; 4 = expert). To support deployment in routine care, the rater is provided with the rationale, description, considerations for scoring, as well as sample language or questions that help to indicate competent execution of the skill in an installation and coaching session.

### Step 4: Monitor Fidelity in the Intervention

#### Adherence Assessment

As emphasized above, our approach to collecting behavioral data on which to base fidelity feedback was intended to balance pragmatics and clinical best practices for fidelity assessment (Ginsburg et al., 2021). The adherence checklist is completed by the mHSS directly; the mHSS self-reports the presence or absence of relevant clinical activities (e.g., meeting frequency, documentation, communication with clinical team) with three clients from the previous 6-month assessment period. It can typically be completed within 10–15 min and can be self-scored and co-reviewed with a clinical supervisor. Due to the ease of administration and scoring and the fact that the adherence checklist collects data that is relevant to the dose of the mHealth intervention, clinical sites may consider embedding the adherence self-check process into the clinical workflow at regular intervals (e.g., quarterly) for continuous quality monitoring.

#### Competency Assessment

Clinician self-reported competencies are often misaligned with observer ratings (Becker-Haimes et al., 2022). We therefore selected an observer-rated assessment of core competencies. To maintain consistency with our objective to achieve a pragmatic FMS that can support rapid proliferation of integrated mHealth interventions in real-world settings, we employed behavioral rehearsal using standardized simulated patient scenarios (Cross et al., 2007). Behavioral rehearsal is a well-known and highly effective training method (Beidas & Kendall, 2010) that has more recently been proposed as a viable fidelity proxy (Beidas et al., 2014; Dorsey et al., 2017). Behavioral rehearsal can be conducted remotely via phone or videoconference, making it a flexible analogue for assessing clinical and technical competencies. We integrated the assessment of core clinical competencies with this video-based behavioral rehearsal into the standardized mHSS training and recertification process (as described in Buck et al., 2022). At the conclusion of training activities, the mHSS interacts with a standardized patient played by the mHSS trainer. Standardized mHealth call scenarios include technology-related barriers and client-related barriers.

Throughout the FOCUS implementation trial, we assessed fidelity using the FOCUS-FMS at the conclusion of mHSS training and every 6 months thereafter. Depending on the complexity of the intervention, individual needs of the interventionist, or clinical quality assurance practices observed by the agency deploying the intervention, behavioral rehearsal can be repeated at fixed intervals to ensure mHSS are meeting performance expectations. Repeat monitoring of adherence and competencies can minimize drift in the delivery of the mHealth intervention and promote integration with other clinical services and processes. In situations where a minimum score reflective of basic competency is not achieved, remediation can be facilitated through supervision and/or training.

### Step 5: Use the Fidelity Ratings in Analyses

The final step associated with fidelity measurement development is the determination of how to use fidelity ratings in the analysis of the outcome data. We consider process data to also be of value here given the broader objective of enhancing uptake and sustainment of mHealth in routine care. As fidelity monitoring is relevant to both practice and research, we describe the application of mHealth-FMS data to each here, in turn.

#### Practical Application of mHealth Fidelity Data

We propose that fidelity is assessed using the FOCUS-FMS at the conclusion of mHSS training and every 6 months thereafter. Depending on the complexity of the intervention, individual needs of the interventionist, or clinical quality assurance practices observed by the center deploying the intervention, behavioral rehearsal can be repeated at fixed durations to ensure mHSS are meeting performance expectations. Repeat monitoring of adherence and competencies can minimize drift in the delivery of the mHealth intervention and promote integration with other clinical services and processes. In situations where a minimum score reflective of basic competency is not achieved, remediation can be facilitated through supervision.

Consistent with our aim of real-world uptake, the competency items may be omitted or administered less frequently than the adherence checklist. The adherence checklist can be quickly scanned by supervisors or administrators to facilitate mHealth clinical and practice facilitation quality improvement and accountability, reinforce effective practices, or identify challenges with mHealth administration. Once a mHSS meets minimum fidelity standards, sites may wish to establish prolonged intervals between competency assessments. Competence ratings can also be used to facilitate identification of mHealth internal trainers, rectify drift, identify individual and organizational strengths and weaknesses in mHealth-related competencies, and demonstrate adoptions and sustainment of the mHealth intervention.

#### Research Application of mHealth Fidelity Data

In research contexts, these data permit a nuanced exploration of the relationships between adherence, competence, and individual and organizational outcomes. Fidelity is underreported in the dissemination of empirical validations of psychological treatments (Perepletchikova et al., 2007), a shortcoming that diminishes important context in discerning trial findings as well as research replication and real-world application of the treatment. Mohr and colleagues (2017) have called for a more efficient research trial design to accelerate the pipeline of digital mental health development, research, and real-world implementation, in which intervention fidelity may be explored as implementation outcomes-of-interest and can support moderation and mediation analyses of the clinical effectiveness of the digital intervention. In the FOCUS Hybrid implementation-effectiveness trial, fidelity data will be compared across mHSS facilitation conditions, and fidelity data will be assessed as an implementation outcome as well as in analyses exploring fidelity as a potential mediator of client response.

## Discussion

Given the nascency of the deployment of mHealth applications in routine care settings, significant questions remain regarding requisite competencies; approaches to training interventionists in foundational and/or functional competencies; how to assess mHealth competencies; and how to ensure that training and quality assurance processes are acceptable, appropriate, and feasible. In this paper, we build on previous work explicating core competencies for mHealth delivery, proposing approaches to training this new workforce, and enumerating the importance of fidelity standards by describing the development of an mHealth fidelity monitoring system.

**Fig. 2:**
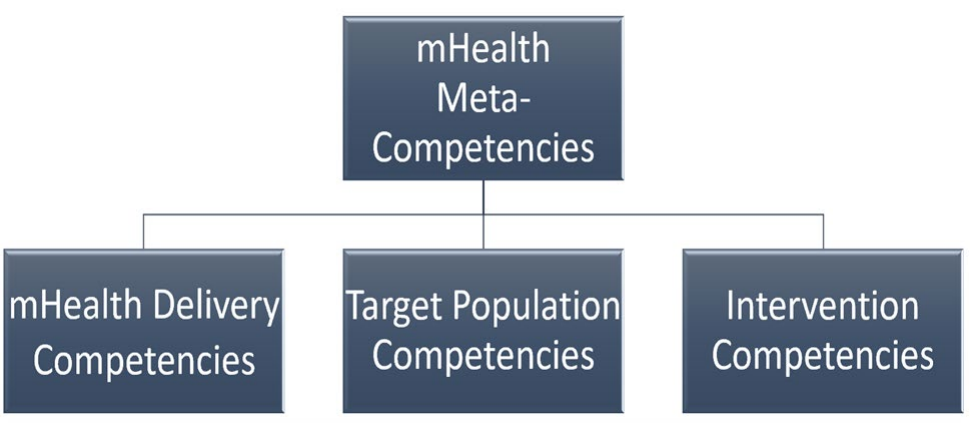
mHealth meta-competency framework template

To date, no integrative competency frameworks have been produced to incorporate the clinical and technical competencies deemed critical to both mHealth and the underlying intervention being delivered through digital delivery (e.g., metabolic monitoring, behavioral activation, smoking cessation). Integration of mHealth in clinical contexts should presume a minimum threshold of foundational competencies in both the underlying intervention and the population for which the mHealth-delivered intervention is intended. We propose that, while previous proposals of mHealth competencies have laid important groundwork, they should be thoughtfully integrated with competencies relevant to the target clinical population and the substratal empirically-supported intervention. A proposed template for this competency framework is depicted in Fig. 2. This approach enables the mHealth interventionist to support both technical and clinical skill development and may be particularly helpful in resource-scarce settings, among populations that require more hands-on support (e.g., clients with cognitive impairment), and/or in circumstances of fragmented or episodic care.

We operationalized the identified meta-competencies for FOCUS to forge a novel mHealth intervention fidelity monitoring system that is a pragmatic option for real-world deployment. By blending empirically-guided mHealth core competencies with recommendations for fidelity development proposed by the Treatment Fidelity Workgroup of the NIH Behavior Change Consortium, the tool is intended to ground both the direct service provider and the system of care in which they operate in the core functions, tasks, and needed competencies for mHealth delivery. The mHealth-FMS was designed to be compatible with community health clinic workflows. For instance, the assessment can be conducted with direct observation of live or recorded mHealth phone-based encounters or through a role play with a standardized client. Similarly, the tool can be used as a learning and self-reflection tool by the mHealth support specialist in order to improve adherence and/or competencies related to the mHealth protocol. Finally, the mHealth-FMS can serve as a benchmarking tool for mHealth quality assurance and improvement efforts in order to optimize the likelihood of behavior modification and symptom improvement.

### Limitations

Data collection is currently underway on the use of the fidelity monitoring system described here by digital health interventionists in the hybrid type 3 FOCUS RCT. As such, we are limited in our ability to comment on the feasibility, acceptability, appropriateness, and outcomes associated with the fidelity tool we developed. We are actively recruiting, training, and monitoring new mHSS as part of the trial. We have successfully implemented the fidelity monitoring system with 30 mHSS across 25 community mental health centers to date. Based on the progress of the ongoing implementation trial, the system appears both feasible and acceptable. mHSS are able to complete the adherence checklist in less than 10 min; the behavioral rehearsal is completed in less than 45 min, inclusive of scoring. The monitoring system produces variability in scoring across multiple specialists and has supported identifying mHSS with higher or lower levels of mHealth competency. Feedback on the use of the system indicates that it provides opportunities to enhance implementation workflows (e.g., additional leadership meetings, improved clinician education) as well as clinical coaching (e.g., utilizing agendas, eliciting goals from clients). Future investigations will analyze the association between fidelity performance and both implementation and clinical outcomes collected as part of the RCT.

We assert that digital interventionists represent a new segment of the behavioral health workforce. This perspective, which has yet to be empirically explored, has been informed by the authors’ collective experience in digital mental health intervention development, behavioral health workforce training in empirically-supported psychosocial treatments, behavioral health and digital mental health policy at the state and federal levels, and Dissemination and Implementation science. Specifically, we have personally observed the now well-documented challenges with uptake, adoption, and integration of new interventions in routine practice settings. While these challenges are multifactorial, one factor that is related to the internal context pertains to the increasing demands placed upon clinicians to maximize billable time. Digital health interventionists—particularly those working with clients with SMI and associated challenges (e.g., organization, housing instability)—must be available to engage in activities that are non-reimbursable but critical to adoption and sustainment. For instance, they may need to send prompts to clients, spend time teaching clients how to charge their device, monitor data among patients, create data digests that can be shared with other members of the clinical teams, or provide targeted recommendations to other clinicians based on app-derived data. Although it may be the case that practitioners serving other patient populations could absorb these additional tasks, we recommend that the tasks are supported by a dedicated interventionist who can facilitate good uptake at the client, practitioner, and system levels. Whether this can be demonstrated remains to be determined through subsequent comparative effectiveness trials.

### Future Directions

Unlike evidence-based medical and psychosocial interventions, mHealth interventions are disproportionately generated by private industry. As such, the science that will amass around these interventions will depend as much on practice-based evidence as they will on evidence-based practice. Community-based research is needed to enhance our understanding of how individual (patient and practitioner), system, and contextual factors impact intervention adoption and use. Such factors can guide indicated adaptations, which will necessarily affect how we conceptualize and approach fidelity assessment (Carvalho et al., 2013). For instance, a client-facing fidelity assessment may support behavioral shaping of clients’ use of prescribed mHealth interventions, thereby facilitating improved outcomes of the intervention target. Moreover, because use data is collected passively and feedback can easily be automated into the client interface, quality assurance can be built into the intervention (Hermes et al., 2019; Reger et al., 2013).

Future research should assess the relationship between mHealth adherence, competence, and patient outcomes. Scholars have not yet established whether there is a minimum fidelity threshold needed to produce sufficient clinical changes for target clients. Borrelli et al. (2005) suggested a criterion of 80% based on a review of the literature, but such a threshold is neither empirically established nor uncontested. As mHealth-FMS begin to be integrated in mHealth-delivering clinics, concurrent collection of fidelity data alongside clinical outcome data may inform minimum thresholds for client-facing and system-facing activities as well as recommended dosage (Sanetti & Kratochwill, 2009). Finally, whereas fidelity tools are typically intervention- or program-specific, the mHealth fidelity tool we developed is intended to generalize to mHealth interventions intended for other clinical targets (e.g., diabetes management, sleep hygiene) that are intended to be facilitated by a clinical support person. The extent to which this assumption is accurate bears investigation.

Finally, it is critically important to evaluate the extent to which adoption of a fidelity monitoring practice can enhance both service delivery and institutional absorption. As mHealth interventions become woven into the fabric of healthcare, practitioners will play important roles in enabling mHealth uptake, engagement, and outcomes that are comparable to those demonstrated in the empirical literature. Preparing the healthcare sector to facilitate digital interventions requires both training and quality monitoring to enhance the effectiveness of the evidence-based mHealth interventions being implemented (Buck et al., 2022; Hermes et al., 2019). Moreover, as the empirical research on human-assisted mHealth interventions for diverse patient populations, settings, and applications proliferates, methodological practices to monitor and enhance treatment fidelity are needed to enhance internal validity, reliability, and statistical power (Bellg et al., 2004). We capitalized on opportunities afforded by a NIMH-funded hybrid implementation-effectiveness trial to delineate an integrated competency framework for human-supported mHealth and developed an mHealth-FMS that could be both empirically and clinically useful. The future of integrated mHealth requires continued expedition into the strategies that will facilitate optimal implementation and intervention outcomes.
